# Waste not, want not: A bio-economic impact assessment of household food waste reductions in the EU

**DOI:** 10.1016/j.resconrec.2019.04.016

**Published:** 2019-07

**Authors:** George Philippidis, Martina Sartori, Emanuele Ferrari, Robert M'Barek

**Affiliations:** a European Commission, Joint Research Centre (JRC), Seville, Spain; b Aragonese Agency for Research and Development (ARAID), Centre for Agro-Food Research and, Technology (CITA), Agrifood Institute of Aragón (IA2), Government of Aragón, Zaragoza, Spain

**Keywords:** Food waste, CGE, EU, Households’ food waste, Packaging, Costs of reducing food waste

## Abstract

Halving per capita food waste and reducing food losses along production and supply chains is one of the objectives fixed by the United Nations among the Sustainable Development Goals (SDGs). This study employs a system-wide modelling approach, to simulate economic impacts of households' food waste reduction in 2030, focusing on the European Union. The paper provides market, food security, biophysical and environmental indicators resulting from food commodity specific waste reductions by EU households. The scenarios explicitly account for associated costs paid by food manufacturers derived from adjustments in the packaging, labelling and logistics. The model shows, under a series of market assumptions, a possible increase in household savings, a fall in agri-food production and a minor negative macroeconomic impact due to household food waste reductions. On the other hand, the effects on environmental indicators such as land usage, GHG emissions and water abstraction are positive. Future research on several aspects of the food waste topic is needed to better integrate all economic aspects.

## Highlights

• Assessment of food waste reduction impact needs to include economic and biophysical indicators.
• Economy-wide models as proposed in this paper can account for all market and non-market impacts of reducing food waste.
• Reducing households' food waste increases savings; it decreases agri-food production and to a minor extent GDP.
• Environmental indicators are improving as EU land use, water abstraction and greenhouse gas emissions decline.

## 1 Introduction

As the world faces the multiple challenges of increasing population growth, climate change and land use pressures, there is an ever increasing urgency on the part of policy makers to devise realistic sustainable solutions. Traditionally seen as an ethical responsibility, food waste reductions represent a legitimate part solution to lessening the misappropriation of economic resources and their resulting negative environmental externalities. For example, the FAO (2014) estimates that the global annual cost associated with food waste amounts to 2.6 trillion USD, equivalent to 3.3% of global GDP. The FAO (2013) also estimates the non-market benefits of food waste reductions, in terms of global water savings of approximately 250 km³ of water each year, whilst other commentators refer to the avoidance of harmful fertilisers and the alleviation of cropland pressures (Kummu et al., 2012; Vanham et al., 2015). Finally, Monier et al. (2010) estimate that at least 170 Mt of CO_2_-equivalent are emitted because of food waste (around 3% of total greenhouse gas (GHG) emissions in the European Union -EU-), out of which 78 Mt are due to households' food waste.

For these reasons, limiting food waste is firmly legitimised as a key priority within policy circles. This observation is particularly pertinent when examining the Sustainable Development Goals (SDGs) formalised in September 2015 by the United Nations (UN, 2015). Under Goal 12, defined as 'responsible consumption and production', target 12.3 makes explicit reference to "halving per capita global food waste at the retail and consumer levels and reducing food losses along production and supply chains, including post-harvest losses" (UN, 2015, pp22). In the EU, under the mantra of the Circular Economy Package, a programme of zero waste was launched in 2014 (European Commission, 2014), which in 2018, was followed up by revised EU Waste Legislation (European Union, 2018) which calls on member states to actively pursue the monitoring and reducing of food waste at each stage of the supply chain.

Recognition of the importance of food waste is also reflected in the growing academic literature, which examines (inter alia) its causes (Schanes et al., 2018), monitoring (Corrado and Sala, 2018) and management (Cristobal et al., 2018). Studies in the economic literature which focus on the short-run (Campoy-Munoz et al., 2017) and medium-run (Rutten et al., 2013a) quantitative impacts of food waste reductions, typically employ a systems-wide macroeconomic simulation approach that explicitly recognises the direct impacts along different stages of the food chain and the resulting ripple effects for the broader macro-economy.

Following employing an approach more akin to Rutten et al. (2013a), the aim of the current paper is also to examine the medium-term market impacts arising from EU food waste reductions. Due to a paucity of detailed data estimates of food waste, the focus is confined to EU household food waste reductions, which according to Stenmarck et al. (2016) account for over half of food waste. The objective of the study is to follow a time horizon to 2030, compatible with the SDG proposals, whilst it also seeks to establish the hitherto ignored importance of the dimension of supply side adjustments in labelling, packaging and logistics in the food chain as a key measure to achieve associated household food waste reductions. A further onjective is to extend the current market-, food security and land use indicators arising from food waste reductions presented in Rutten et al. (2013a,2013b), to encompass additional biophysical and environmental indicators.

Consistent with previous studies, the results show that household food waste reductions generate falls in EU agri-food production, whilst the food price effect is indeterminate, based on the opposing agri-food supply and demand forces related to household food waste reductions and rising unit costs of production. Elsewhere, EU food security is shown to improve, whilst the magnitude of the biophysical and environmental benefits are enumerated, in terms of reduced land use pressures, contractions in water abstraction and greenhouse gas reductions.

The rest of this paper is structured as follows. Section two provides a discussion of the literature. Section three outlines the material and methods. Sections four and five present the results and provide some discussion. Section six concludes.

## 2 Related literature

The measurement of the quantity and value of food waste remains a contentious issue (Bellemare et al., 2017), although Stenmarck et al. (2016) note that estimates for the EU in 2012 place it at approximately 81 million tonnes (Eurostat, 2017) and 87.6 million tonnes (Stenmarck et al., 2016). The uncertainty behind the scale of the problem is, at least in part, because the definition and measurement of food waste remains problematic (Scherhaufer et al., 2018; Schneider, 2013). In their seminal article, Corrado and Sala (2018) conclude that the measurement of waste streams at European and global scales can vary significantly depending on the choice of methodologies for measurement, the inconsistent usage of definitions such as 'avoidable vs non avoidable' food waste (a sentiment also echoed by Lebersorger and Schneider, 2011) and the reliability of the underlying data. To illustrate this uncertainty, they note that estimates of European per capita food waste ranges between 158 kg – 298 kg per year. In other studies, however, household food waste is estimated at 76 kg per person per year (Monier et al., 2010 based on 2006 data for the EU27) and 92 kg per person per year (Stenmarck et al., 2016 based on 2012 data for the EU28 including edible and inedible).

A further issue is the myriad of economic drivers of household food waste in developed societies (FAO, 2014; Schanes et al., 2018). It is posited (Segrè et al., 2014) that microeconomic theory often fails to capture ‘real’ consumption behaviour arising from non-price factors including poor planning decisions, perceptions of aesthetics and social prestige and the relationship between low purchasing power and low nutrition food choices. The authors also allude to cultural- and lifestyle-factors (i.e., declining culinary knowledge, bad food management), as well as the low ethical-, environmental- and cost perceptions of food waste in societies with highly abundant food at relatively low cost. Indeed, on this latter point, Lusk and Ellison (2017) observe that an individuals' sensitivity to time supersedes food waste concerns, resulting in behaviour to limit trips to the store through bulk buying behaviour.

Other relevant literature examines the role of labelling and packaging innovations to help reduce household food waste. Consumers widely misunderstand current labels that are strongly associated with food waste. A consumer survey of the USA reports that 37% of consumers always or usually discard food when it is close to the "best before" package date (Neff et al., 2019). Williams et al. (2012) estimate food losses due to issues with packaging to be 20–25% of household food waste. They confirm that bulk packs and date labelling are major factors. It is therefore logical to deduce that innovations in food packaging and labelling (e.g., re-closable packs, smaller and subdivided packs, more detailed label advice, time–temperature indicators and control) are key drivers to reduce household food waste. Verghese et al. (2015) suggest that clearer labelling advice could be realised at limited additional cost to the food retailer, whilst also postulating that the costs associated with smart tags and thermal sensor technologies, may be (partially) mitigated by associated benefits in terms of reduced food waste, whilst Schanes et al. (2018) allude to other unit cost reducing benefits associated with improved packaging solutions due to fiscal incentives to food producers.

Economic modelling representations of food waste reductions in the literature are, by necessity, simplified representations of the multi-layered mechanics which drive household behaviour discussed above. Indeed, the focus is more toward the resulting market impacts, which are ultimately influenced by plausible data estimates of food waste, elasticities of demand and supply and the market interactions between consumers and producers (Bagherzadeh et al., 2014). Moreover, the repercussions of reducing household food waste extend beyond the direct impacts on agriculture and food activities, to include the ripple effects on upstream input markets (i.e., feed, fertiliser usage, land and labour), as well as food security benefits arising from reduced food import dependence. For this reason, FAO (2014), supported by its High-level Panel of Experts on Food Security and Nutrition, proposed a systemic general equilibrium model framework. Examples in the relevant literature employ macroeconomic simulation models such as fixed-price social accounting matrices (SAM) (e.g., Campoy-Munoz et al., 2017) or flexible-price computable general equilibrium (CGE) representations (Britz et al., 2014; Rutten et al., 2013a; Rutten and Verma, 2014; Rutten et al., 2015; Rutten and Kavallari, 2016).

In the context of *household* food waste, the literature is narrower. Campoy-Munoz et al. (2017) model first-order reductions in household consumption to mimic uniform food waste reductions of 15% across all consumed food commodities in Germany, Poland and Spain. Their main results show decreases in agricultural production, lower employment and a GDP loss of between -1.21 and -2.15%. Employing secondary data estimates of food waste by food product categories, Rutten et al. (2013a) examine 30%, 40% and 50% rate reductions in EU-wide household food waste over the period 2012−2020. The authors draw the same qualitative conclusions as in Campoy-Munoz et al. (2017), whilst also showing that household savings increase by between 92, 123 and 153€ per capita under each of the aforementioned reductions, respectively. Neither study considers the social costs associated with this behavioural shift, although Britz et al. (2014) employ a conceptual modelling approach to tentatively explore the labour-leisure household trade-off as food waste reductions associated with increased home preparation reduces leisure time. Their study reveals that when the household utility function explicitly recognises the time cost associated with reducing food waste, the resulting impact on production and prices can change substantially.

Following the work of Rutten et al. (2013a), this paper revisits the issue of household food waste reductions in the EU, employing a multiregional CGE simulation model, with a highly comprehensive baseline scenario to 2030 which captures an array of economic, biophysical and policy drivers (see next section). As in Rutten (2013a), food waste reduction scenarios draw on recent food commodity-specific secondary data estimates of household food waste (see next section). In recognition of the aforementioned literature, a novelty of this study is that it captures hitherto ignored agri-food supply side drivers induced by associated compliance cost increases (e.g., improved labelling schemes to remove misinterpretations; technological improvements to identify microbial risks; improved re-sealable packaging to reduce water loss; interactive films, or new technologies to reduce oxygen degradation). Additionally, the broader (non-economic) repercussions of EU household food waste reductions are examined in this study. More precisely, this paper benefits from additional modelling refinements and accompanying satellite data to encompass biophysical (water abstraction) and environmental (greenhouse gas) indicators as well as the social indicator of employment (in number of persons).

## 3 Material and methods

### 3.1 Methodology

To enumerate the impacts of household food waste reductions, this study employs the Modular Applied GeNeral Equilibrium Modelling Tool (MAGNET) (Woltjer and Kuiper, 2014). Given its high degree of modelling flexibility (see below), examples of peer reviewed MAGNET impact assessments have appeared on issues relating to land-use change (Schmitz et al., 2014); EU domestic support (Boulanger and Philippidis, 2015); biofuels policy (Banse et al., 2011); food security (Rutten et al., 2013b) and climate change (Nelson et al., 2014).

At its core, the MAGNET model is in the family of neoclassical multi-region CGE models and is based on the global database (Aguiar et al., 2016) and model (Corong et al., 2017) developed by the Global Trade Analysis Project (GTAP) network. In its tenth incarnation, the GTAP data covers 140 regions and 57 tradable commodities, of which twenty are related to primary agriculture and food. The macroeconomic component is based on national input-output accounts with detailed coverage of intermediate and final purchases at agents' and purchasers' prices. These core tables are connected by data on gross bilateral trade flows supplemented by trade distortions (i.e., tariffs/subsidies) and transport costs.

The model core follows the standard conventions of neoclassical optimising behaviour (cost minimisation, utility maximisation) on the part of agents (firms, households, investors and government). Production structures are typified by constant returns to scale technologies and perfectly competitive market structures. Market clearing conditions ensure that supply and demand yield prices which generate a 'unique' general equilibrium solution in all 'n' markets whilst further accounting conventions ensure parity between the macro relationships, namely the income-, expenditure- and output-flows within the circular flow of income. Fixed regional savings rates are assumed, whilst the typical macro closure enforces the rule that the capital- and current-accounts ensure a net zero balance of payments.

The MAGNET model is an advanced derivative of this core structure. From a data perspective, there are additional sector splits with greater detail regarding (inter alia) feed and energy (both bio- and renewable), which allows for an improved and differentiated treatment of primary agricultural nested production technologies in crop and livestock sectors. Moreover, the modular structure of MAGNET allows the user to switch on/off non-standard modelling features which are pertinent to the focus of the study. Thus, to more explicitly capture the resulting market trade-offs between feed, food, fuel and material uses of biomass in response to reductions in household food waste, this variant of MAGNET includes additional modules to explicitly capture the sector specificity of agricultural labour and capital, heterogeneous land transfer between different agricultural activities and endogenous agricultural land supply. To provide greater policy detail within the study, further modules explicitly capture the EU's Common Agricultural Policy (Boulanger and Philippidis, 2015), conventional biofuels policies (Banse et al., 2011); and environmental emissions limits (Burniaux and Truong, 2002). A final pertinent improvement is the treatment of food demand patterns over medium term time frames, which relies on downward endogenous adjustments to calibrated household income elasticity parameters in regions with rising per capita real incomes (Woltjer and Kuiper, 2014).^1^

### 3.2 Aggregation and scenario design

In this study a medium-term status quo baseline is implemented from 2011 to 2030 over three time periods (2011–2015; 2015–2020; 2020–2030) which follows expected macroeconomic- (real GDP, population), biophysical- (land productivities) and energy- (fossil fuel prices, energy consumption and production trends) trends. Furthermore, attention is given to environmental- (region-wide GHG reductions) and biomass-policies (Common Agricultural Policy, bioenergy support, trade liberalization) based on respected secondary data sources. A detailed discussion of all of these assumptions, the relevant data sources and the modelling approach, is available online in Philippidis et al. (2018).

The chosen model aggregation (see Table A1 of the Appendix) includes the full coverage of sectors within the GTAP database, which accounts for eight crops sectors, four livestock sectors, one dairy sector, two meat sectors (red and white meat) and a further four food processing sectors. In addition, non-standard agricultural input sectors of animal feeds and fertiliser are explicitly recognised. The commodity disaggregation also covers alternative sources of biomass in biomaterials and bioenergy, substitute fossil technologies, and several manufacturing and services composite sectors (Philippidis et al., 2018). In large part, the richer representation of commodity splits permits a fuller representation of differentiated technologies for livestock (feed inputs) and cropping (fertiliser usage) activities. Since reliable detailed commodity-by-commodity food waste data is only available for the EU aggregate, this is represented as a single composite region in the model. As the principle suppliers of biomass, the non-EU regions are split into the continents of the rest of the European neighbourhood, North and South America, Africa, and Asia and Oceania.

To motivate household food waste reductions, official estimates produced by the Joint Research Centre (JRC) of the European Commission^2^ are employed. The authors performed a mass flow analysis of the European food system in 2011 (same as the benchmark year of the model). Food waste was mapped and quantified, generating flows across the food supply chain with a breakdown for each of the four stages of the supply chain, i.e. primary production; processing; retail; and consumption (both at the household and food service sector level), for different food groups. Sources of data include FAO statistics (commodity balance sheets and trade data); Eurostat data (trade and production of manufactured products – Prodcom); studies from the literature (to obtain food waste coefficients), and EU food consumption statistics. Measured in million tonnes of wet matter, Table 1 shows the resulting percentage of EU household consumption by commodity which is wasted (both avoidable and non-avoidable waste), where vegetables (26%) fruits and meat (19%) are found to exhibit the highest waste ratios^3^ .

**Table 1:** Estimates of EU household waste by food commodity.

COMMODITIES	% HH food waste
Fruits	19%
Vegetables	26%
Sugar	12%
Cereals (including bread and pastry)	12%
Fish	12%
Meat	19%
Dairy	8%

Thus, comparing with the baseline, four food waste reduction scenarios (labelled 25_01, 25_05, 50_01 and 50_05, see Table 2) are modelled reflecting two fundamental agri-food market drivers. Motivated by target 12.3 of the SDG, a demand driver reflects falling aggregate EU household food consumption from reductions in food waste corresponding to 50% of the estimates in Table 1. In recognition of the uncertainty in measuring food waste, a more moderate 25% food waste reduction scenario is also explored, which is also comparable with the lower bound threshold in Rutten et al. (2013a). The modelling of food waste reductions by commodity category is performed using household budget share shifters that adjust endogenously to meet targeted household consumption reductions.

**Table 2:** Food waste scenarios.

	Compliance costs 1%	Compliance costs 5%
Food waste reduction 25%	25_01	25_05
Food waste reduction 50%	50_01	50_05

The second driver is supply related, arising from hypothesised per unit compliance costs to trigger behavioural changes in European household food-consumption, as discussed in Section 2. To the best of our knowledge, there are no empirical estimates on the magnitude of said costs. Notwithstanding, evidence from our trawl of the literature in Section 2 and on the basis of expert opinion,^4^ we assume that the compliance costs vary between a lower and upper bound of 1% and 5% of the value of the relevant sales flows. On intra-EU transactions, compliance costs calculated from intra-community sales of each agri-food commodity, are implemented in the cost function of the relevant EU agri-food industries as per unit cost increases on the use of services inputs. On EU’s extra-community imports, these costs are imposed at the EU border as a corresponding per unit import cost increases within the Armington function. Given a lack of reliable data on commodity-specific food waste for the rest of the world, it is assumed that non-EU countries do not implement corresponding food waste cutting initiatives. As a result, EU extra-community exports of food do not face any reciprocal compliance costs.

## 4 Results

### 4.1 EU market impacts

**Fig. 1:**
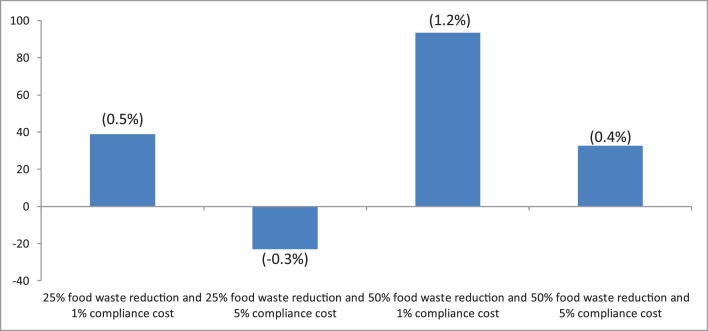
Change in per capita household expenditure (EUR/per year) versus baseline (2020–2030 change). Share over household expenditure in brackets.

With a highly localised market (i.e., agri-food) impact within the entire EU economy, the macroeconomic impacts are, as expected, relatively muted. The impact on real GDP, although negative in all scenarios, ranges between about -0.1% and -0.5% (not shown). Fig. 1 shows the yearly change on per capita household savings (€/year) in the year 2030, compared to the baseline. The reduction in household food consumption reflecting reduced waste dominates the compliance cost effect (higher per unit prices), resulting in household savings in most scenarios. Fig. 1 shows that per capita savings reach the highest value of 93€ under the 50_01 scenario (highest food waste reduction, lowest compliance cost) although result in a household expenditure increase of 23€ under the 25_05 scenario (lowest food waste reduction, largest compliance cost). Compared to other studies (e.g., Rutten et al., 2015; Waste and Resources Action Programme - WRAP)^5^, our estimates are less optimistic due to the price inflationary impact of the compliance costs.

**Fig. 2:**
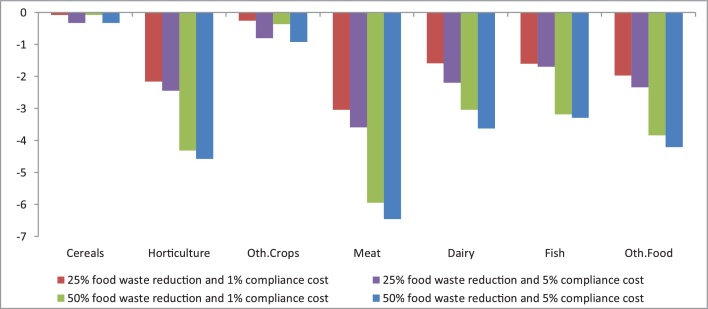
Percentage change in EU production trends compared to the baseline (2020–2030 change).

Consistent with previous studies (Rutten et al., 2013a; Campoy-Munoz et al., 2017), a contraction in EU domestic food demand together with per unit cost increases to agri-food supply chains results in unambiguous production falls in all affected agri-food activities compared to the baseline (Fig. 2). With high estimates of food waste in both food product categories (see Table 1), the largest production decreases are observed for horticulture and meat. The decrease in dairy and fish is also notable, since a large sales share accrues to household final demand. The small decrease in cereals output suggests that final demand is relatively inelastic.

**Fig. 3:**
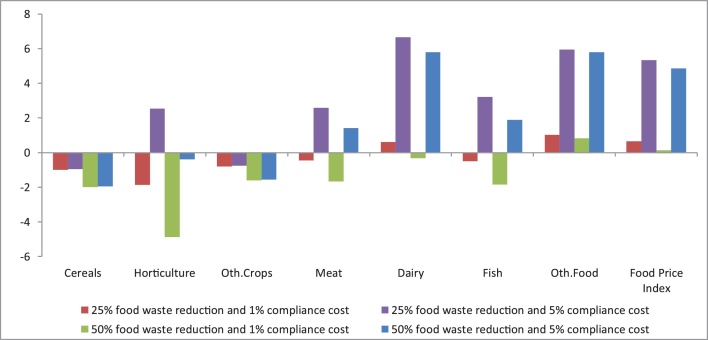
Percentage change in EU food prices compared to the baseline (2020–2030 change).

The effect on price is more ambiguous, despite the food price index (Fig. 3) increases under all scenarios. In fact, the impact on prices differs across agri-food sectors, since price changes are a net effect of the presence and magnitude of the two opposing drivers of demand (food waste reductions) and supply (compliance costs) and their associated elasticities (i.e., price sensitivities) in each agri-food market. The demand shifting effect dominates for the cereals and other crops sectors so the price change is negative. For horticulture, meat and fish, the demand effect also dominates under the scenarios with 1% compliance costs assumed, although at the higher assumed compliance cost of 5%, the price effect is generally positive. Examining the food price index, the compliance cost effect is the stronger driver resulting in general price rises of approximately 5%, whilst there are particularly marked impacts for the dairy commodities (between 6–7% price rises).

**Fig. 4:**
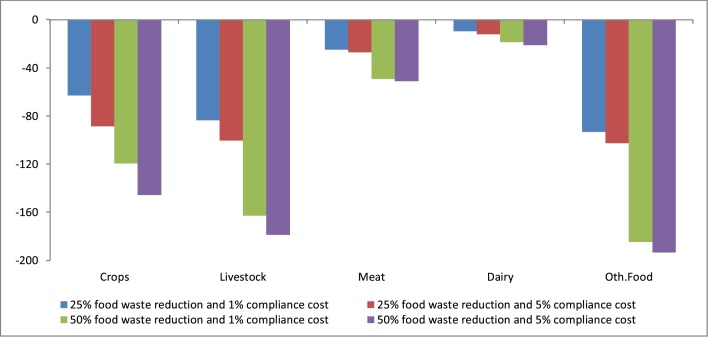
Change in agricultural employment (thousands of heads) compared to the baseline (2020–2030 change).

As expected, the contraction in agriculture and food production inevitably leads to agri-food job losses compared to the baseline (Fig. 4). Meat waste reduction reduces livestock employment by up 179,000 jobs (-4.9%) and meat industry employment by up to 51,000 jobs (-7.3%) compared to the baseline. As a whole, the losses in agricultural jobs amount to 325,000 (-2.9%) and 265,000 in food compared to the baseline (-4.6%). Accordingly, falling demand for agricultural workers (3.8%) depresses real wages by around 3.5% (not shown).

### 4.2 Food security

**Fig. 5:**
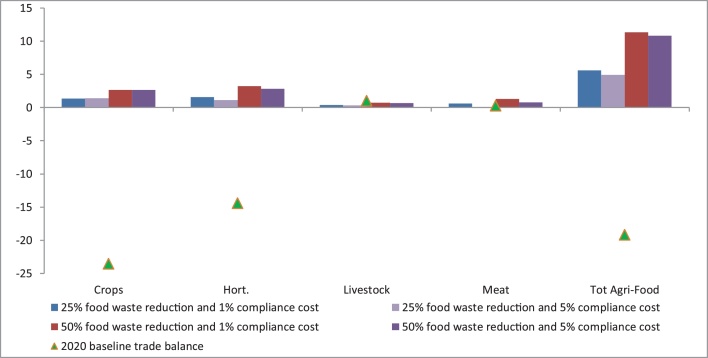
Change in EU agri-food trade balance (exports minus imports, billions of euros) versus baseline (2020–2030 change). Fish and dairy activities not shown as their trade balance is small and the change is irrelevant.

The results of Fig. 5 illustrate the marginal repercussion of the household food waste reduction on the economic agri-food trade balance (i.e., exports minus imports), measured in billions of euros. As noted in Sction 3.2, the food waste cuts are applied to EU household agri-food demand (which is an aggregate of domestically produced and non-EU origin imports) using budget expenditure shifter variables. The compliance costs are imposed on both domestic (i.e., intra-EU) agri-food production and extra-EU imports of agri-food. As a result, in all activities and the whole agri-food sector, EU trade balances improve compared with the baseline, whilst the effect is a positive function of the magnitude of the EU´s household food waste cuts. This observation is especially true for crops and horticulture activities, which drive the change in agri-food trade balance. Alone they represent a significant share (52%) of EU food imports from extra-EU countries. It should, however, also be noted that within a given household food waste reduction (e.g., 25%), as the compliance cost rises (from 1% to 5%), the improvement in the trade balance is lessened as the per unit cost of extra-community imports rises more markedly.

### 4.3 Sustainability

**Fig. 6:**
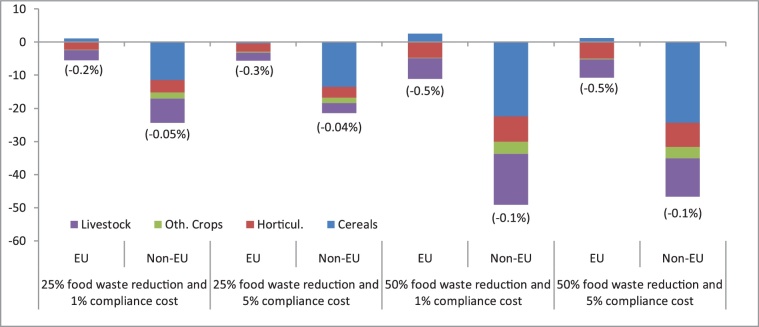
Land use change (thousands of km² and percentages) compared to the baseline (2020–2030 change). Share over land in brackets.

The patterns of land usage are consistent with the production trends (Fig. 6). The EU saves between 4,421 and 9,554 km^2^ of land across the scenarios, equivalent to 0.2% to 0.5% compared to the baseline. The largest contributions to land use savings in the EU typically arise from those sectors where household waste is considered to be higher, such as vegetable and fruits and meat, the latter of which has strong upstream linkages with the live animal and feed sectors.

As a big player in third country agri-food markets, EU households' food waste reductions also have indirect land use change impacts, although relatively moderate. In percentage terms and compared to the baseline, non-EU land use decreases by 0.1% in the 50_01 scenario (highest food waste reduction, lowest compliance cost). In absolute terms, non-EU land use savings are larger (50,000 km^2^). Much of land use savings arise from cereals and livestock activities. On the one hand, reduced imports of non-EU meat may imply less feed demand by livestock sectors in the non-EU regions. The decline in EU meat production (Fig. 2) may also contribute to lessen imported cereals to feed animals. On the other hand, the reduced EU households' demand for domestic and imported animal-derived products implies a reduction of land usage in these activities.

**Fig. 7:**
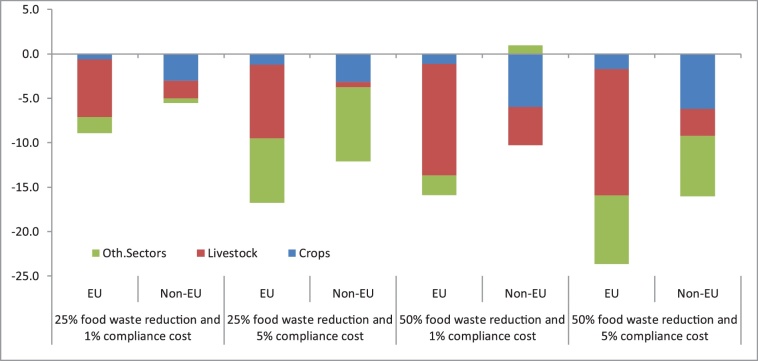
Change in greenhouse gases (million tonnes) compared to the baseline (2020–2030 change).

**Fig. 8:**
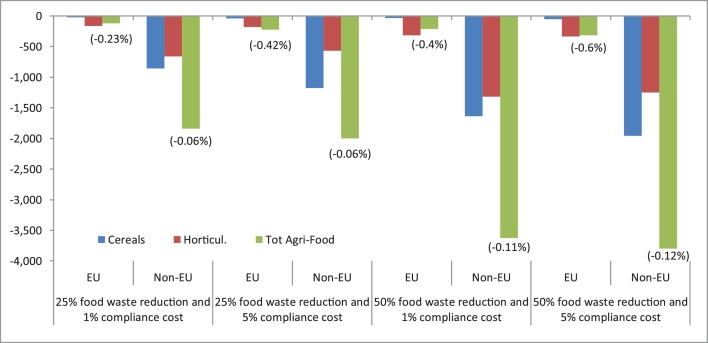
Change in water abstraction for irrigation (millions of cubic meters and percentages) with respect to the baseline. Share over water abstraction in the agri-food sector in brackets.

Among the non-market benefits, reductions in EU household food waste gives rise to positive impacts on reducing GHG emissions (Fig. 7) and water abstraction for irrigation (Fig. 8). Regarding the former, in EU agriculture, the range of emissions reductions is estimated between 7 (-1.6%) and 16 million tonnes (-3.5%). As expected, EU emission reductions are driven by agriculture, but the contribution by non-agricultural activities rises as the compliance cost increases. Livestock, the most emissions intensive sector, is the biggest contributor to this fall, adding up to 60% of the emissions reduction (14.2 million tonnes) under the 50_05 scenario, and driven by the reduction in meat consumption.

As for land, lower EU household demand for imported food translates into lower agricultural production (e.g., lower emissions) in non-EU countries, although with a modest impact (up to -0.1% of emissions reduction in the 50_05 scenario). It is worth noting, however, a couple of interesting results. First, crops activities are always the leading driver in emissions reductions, as the significant reduction in meat production (see Fig. 2) implies a further decrease in the amount of imported feed crops to live animal activities. This observation is confirmed in Fig. 5, where, a notable fraction of the EU trade balance improvement is due to reduced crop imports. Second, the main driver in emissions reductions varies depending on the scenario under consideration. Under the low compliance cost scenarios (25_01 and 50_01), primary agriculture (i.e. livestock and crops activities) accounts for the vast majority of GHG emissions reductions. When the compliance cost increases, the bigger cost impact on services reduces the use of non-agricultural inputs, generating larger emission reductions in the non-agricultural sectors.

Agriculture is the largest water user worldwide, accounting for on average 70% of freshwater withdrawals (FAO, 2011). It is claimed that reducing food waste has also an important positive side-effect on reducing water usage and water pollution from nutrients, pesticides and other contaminants employed in agriculture (Wunderlich and Martinez, 2018). Results shown in Fig. 8 support this observation. For the EU, household food waste reduction generates irrigated abstracted water savings of between -121 (25% and 1% scenario) and -316 million cubic meters (50% and 5% scenario) of water. In percentage terms, the reduction amounts to between -0.2% to -0.6% compared with the baseline. Due to the sheer size of the sector and the assumed reduction in food waste, horticulture is the key driver in EU and non-EU regions. In global terms, water savings approximately amount to 0.1% of global water abstraction in agriculture and are consistent with the expected trends in land usage (Fig. 6).

## 5 Discussion and conclusions

Employing a system-wide bio-economic simulation tool, this study contributes to the debate on the market (economic) and non-market (i.e., biophysical and environmental) implications of EU household food waste reduction. The study employs recent European Commission estimates of physical food waste shares for different food commodities. Comparing to corresponding FAO (2011) estimates, our EU household food waste estimates are noticeably different for cereals (12% instead of 25%, respectively) and meat (19% instead of 11%, respectively). Rutten (2013a) implements the FAO (2011) figures as their starting values, which explains much of the differences in the commodity-by-commodity outcomes compared to the present study.

The analysis shows that, on the one hand, households are expected to save money, while the agri-food sector is expected to shrink in production and employment. The negative macroeconomic impact is consistent with previous studies on this topic. Rutten et al. (2013a) estimate a real GDP fall of -0.1%, which is at the lower end of our range of estimates (-0.1 to -0.5%), because they do not account for the accompanying logistical, labelling and packaging compliance costs met by producers. Employing a linear CGE approach, Campoy-Munoz et al. (2017) record higher negative GDP impacts (-1.21% for Germany, -1.49% for Spain and -2.15% for Poland) due to their assumption of fixed input proportions technology. This assumption implies that producers are unable to change the input mix in response to the market shock, which implies that their impact estimates should be considered as more short-term in nature.

The decrease of market prices and the contraction of all agri-food sectors in the EU is accompanied by a fall in agricultural employment by around 2.5% in Rutten et al. (2013a), which again is below our upper bound estimate of 7% due to the additional compliance cost effect on agri-food producers. A similar line of reasoning also explains the differences in the per capita savings in the EU, where the presence of compliance costs dampens the market price falls arising from household consumption reductions, resulting in smaller budget savings. Indeed, compared with our savings estimate of €93 per capita by 2030, under a 50% food waste cut and 1% compliance cost, the same proportional food waste cut in Rutten et al. (2013a) yields a corresponding saving of 153€ by 2020.

A key insight from this study is that it quantifies the benefits of food waste reductions on non-market indicators within the EU and globally. In terms of land use, our results concur with Rutten et al. (2013a) who also show moderate EU agricultural land use savings (28,940 km²). The more muted effect in our study (9,554 km² or 0.5% of EU agricultural land) is largely attributed to the more inelastic assumption regarding the calibrated agricultural land supply in the EU.

To the best of our knowledge, this is the first attempt to quantify the non-market impacts on greenhouse gases (GHG) and water abstraction arising from food waste reductions within an economic simulation modelling framework. Despite being modest, both indicators exhibit desirable reductions, which is consistent with other studies which extol the benefits of reduced meat intake with reduced water footprints (Vanham et al., 2018) and GHG emissions (Springmann et al., 2018), which is expected to have beneficial spillover effects on ecosystem services.

From a policy perspective, the EU is already taking steps to meet the SDG goal through its Platform on Food Losses and Food Waste (European Commission, 2019), which brings together stakeholders and experts with the express purpose of finding practical solutions for reducing food waste and monitoring progress. Notwithstanding, it has been noted (European Court of Auditors, 2016) that greater coherence of EU policy (i.e., the Common Agricultural Policy, Common Fisheries Policy) with food waste is a priority, whilst improved alignment of regulatory and legal frameworks to facilitate improved food handling and donation networks would also constitute a significant stepping stone. Moreover, our extended range of indicators clearly demonstrate the associated benefits in terms of reduced land and water use pressures, which would provide welcome relief to meeting expected food market access in the face of a growing global population (UN, 2015).

Turning to the caveats of the study, the specification of compliance costs is assumption based, which requires further research. Furthermore, it has been hypothesised that investments to reduce food waste by manufactures could generate some efficiency gains from improved packaging and reductions in product losses which may even offer net benefits to those firms that uptake food waste reduction technologies (ReFED, 2018). Nonetheless, available data does not allow an accurate quantification of these payoffs (Hanson and Mitchell, 2017). In addition, due to statistical and methodological uncertainty, estimates of food waste rates by product category, which are critical drivers of our study, can vary between studies (Corrado et al., 2019). Accordingly, additional research is needed in this area.

A further issue is that imposed reductions in final purchases of food quantities to characterise food waste reductions, does not account for the possibility that consumers may 'trade-up', i.e., consume higher value food products with the resulting household savings. Finally, this study does not include the potential effect that awareness campaigns or other measures might have on food waste reduction. The provision of best-practice information and education may positively influence consumer attitudes, resulting in tangible benefits in terms of reduced food waste. The implementation of any intervention of this type should, however, include the associated cost, the estimation of which is far from straightforward.

With a view to the assessment of the circular economy, (household) waste as a resource for economic activity should also be further considered. As outlined in the recent update of the Bioeconomy Strategy of the European Commission, biodegradable waste (or bio-waste) is potentially an important additional source of biomass (European Commission, 2018, 2018b), either for (inter alia) soil composting or anaerobic digestion for methane (biogas, electricity etc.). Indeed, given the explicit depiction of the circular flow of economic activities within CGE macroeconomic simulation models, an inclusion of these additional activities should be considered as a priority.

## Figures and Tables

**Table A1:** Study disaggregation of commodities and regions.

Commodity Disaggregation (67 Commodities)
**Arable and horticulture (9):** paddy rice; wheat; other grains; oilseeds; raw sugar; vegetables, fruits and nuts; other crops; plant fibres; crude vegetable oil
**Livestock and meat (7):** cattle and sheep; wool; pigs and poultry; raw milk; cattle meat; other meat; dairy
**Fertilizer (1):** fertilizer
**Other food and beverages (4):** sugar processing; rice processing; vegetable oils and fats; other food and beverages
**Other ‘traditional’ bio-based (5):** fishing; forestry; textiles, wearing apparel and leather products; wood products; paper products and publishing
**Bio-mass supply (10):** energy crops; residue processing; pellets; by-product residues from rice; by-product residues from wheat; by-product residues from other grains; by-product residues from oilseeds; by-product residues from horticulture; by-product residues from other crops; by-product residues from forestry
**Bio-based liquid energy (5):** 1 st generation biodiesel; 1 st generation bioethanol; 2nd generation thermal technology biofuel; 2nd generation biochemical technology biofuel; bio-kerosene
**Bio-based industry (4):** lignocelluose sugar; biochemical (fermentation) conversion of sugar biomass to polylactic acid chemicals; biochemical (fermentation) conversion of bioethanol to polyethylene chemicals; thermochemical conversion of biomass to chemicals
**Bio-based and non-bio-based animal feeds (3):** 1 st generation bioethanol by-product distillers dried grains and solubles (ddgs); crude vegetable oil by-product oilcake; animal feed.
**Renewable electricity generation (3):** bioelectricity; hydroelectric; solar and wind
**Fossil fuels and other energy markets (10):** crude oil; petroleum; gas; gas distribution; coal; coal-fired electricity; gas-fired electricity; nuclear electricity; electricity distribution; kerosene
**Other sectors (6):** chemicals, rubbers and plastics; other manufacturing; aviation; other transport; food services; other services
**Regional Disaggregation (6 Regions):**
European Union (EU), Rest of Europe (RestEurope); North America (NoAmerica); Central and South America (SouandCentAme); African continent (Africa); Rest of the world (ROW).

## Footnotes

1 As a result, in regions/countries where real incomes are rising rapidly (i.e., China, India, Mercosur), a more realistic rise in food demands (vs. the standard GTAP treatment) moderates pressure on food prices, and by extension biomass prices and land rents.

2 Official data estimates were received though personal communication (September 20, 2018) with Carla Caldeira, Sara Corrado and Serenella Sala, European Commission-Joint Research Centre, Ispra, Italy, Directorate D (Sustainable Resources), Bioeconomy Unit.

3 Rutten et al. (2013a) follow FAO, (2011) calculations according to which the highest waste share lay in cereals (25%) followed by fruits and vegetables (19%) and fish and meat (11%), while Campy-Munoz et al., (2017) adopt an homogenous reduction over commodities assuming that at the household, food waste represents 25% of food purchased (by weight), of which 60% could be avoidable; therefore, the percentage of avoidable waste is 15%.

4 From personal exchanges with the relevant experts in the Directorate General of Agriculture and Rural Development of the European Commission.

5 Rutten et al. (2015) show that by reducing food waste in consumption, EU consumers could save US$228 per capita per year in 2020. The financial savings for households from throwing away less food were estimated by WRAP as €14bn per year in the UK, or an average €233 per person per year. More information is available at: http://www.wrap.org.uk/
